# Response variability in Attention-Deficit/Hyperactivity Disorder: a neuronal and glial energetics hypothesis

**DOI:** 10.1186/1744-9081-2-30

**Published:** 2006-08-23

**Authors:** Vivienne A Russell, Robert D Oades, Rosemary Tannock, Peter R Killeen, Judith G Auerbach, Espen B Johansen, Terje Sagvolden

**Affiliations:** 1 Department of Human Biology, Faculty of Health Sciences, University of Cape Town, Anzio Road, Observatory 7925, South Africa; 2 University Clinic for Child and Adolescent Psychiatry, Virchowstr. 174, 45147 Essen, Germany; 3 Research Institute of The Hospital for Sick Children, University of Toronto, Canada; 4 Department of Psychology, Arizona State University, Tempe, AZ 85287-1104, USA; 5 Department of Behavioural Sciences, Ben-Gurion University, Beer Sheva, 84105, Israel; 6 Department of Physiology, University of Oslo, N-0317 Oslo, Norway

## Abstract

**Background:**

Current concepts of Attention-Deficit/Hyperactivity Disorder (ADHD) emphasize the role of higher-order cognitive functions and reinforcement processes attributed to structural and biochemical anomalies in cortical and limbic neural networks innervated by the monoamines, dopamine, noradrenaline and serotonin. However, these explanations do not account for the ubiquitous findings in ADHD of intra-individual performance variability, particularly on tasks that require continual responses to rapid, externally-paced stimuli. Nor do they consider attention as a temporal process dependent upon a continuous energy supply for efficient and consistent function. A consideration of this feature of intra-individual response variability, which is not unique to ADHD but is also found in other disorders, leads to a new perspective on the causes and potential remedies of specific aspects of ADHD.

**The hypothesis:**

We propose that in ADHD, astrocyte function is insufficient, particularly in terms of its formation and supply of lactate. This insufficiency has implications both for performance and development: **H1) **In rapidly firing neurons there is deficient ATP production, slow restoration of ionic gradients across neuronal membranes and delayed neuronal firing; **H2) **In oligodendrocytes insufficient lactate supply impairs fatty acid synthesis and myelination of axons during development. These effects occur over vastly different time scales: those due to deficient ATP (H1) occur over milliseconds, whereas those due to deficient myelination (H2) occur over months and years. Collectively the neural outcomes of impaired astrocytic release of lactate manifest behaviourally as inefficient and inconsistent performance (variable response times across the lifespan, especially during activities that require sustained speeded responses and complex information processing).

**Testing the hypothesis:**

Multi-level and multi-method approaches are required. These include: 1) Use of dynamic strategies to evaluate cognitive performance under conditions that vary in duration, complexity, speed, and reinforcement; 2) Use of sensitive neuroimaging techniques such as diffusion tensor imaging, magnetic resonance spectroscopy, electroencephalography or magnetoencephalopathy to quantify developmental changes in myelination in ADHD as a potential basis for the delayed maturation of brain function and coordination, and 3) Investigation of the prevalence of genetic markers for factors that regulate energy metabolism (lactate, glutamate, glucose transporters, glycogen synthase, glycogen phosphorylase, glycolytic enzymes), release of glutamate from synaptic terminals and glutamate-stimulated lactate production (SNAP25, glutamate receptors, adenosine receptors, neurexins, intracellular Ca^2+^), as well as astrocyte function (α_1_, α_2 _and β-adrenoceptors, dopamine D1 receptors) and myelin synthesis (lactate transporter, Lingo-1, Quaking homolog, leukemia inhibitory factor, and Transferrin).

**Implications of the hypothesis:**

The hypothesis extends existing theories of ADHD by proposing a physiological basis for specific aspects of the ADHD phenotype – namely frequent, transient and impairing fluctuations in functioning, particularly during performance of speeded, effortful tasks. The immediate effects of deficient ATP production and slow restoration of ionic gradients across membranes of rapidly firing neurons have implications for daily functioning: For individuals with ADHD, performance efficacy would be enhanced if repetitive and lengthy effortful tasks were segmented to reduce concurrent demands for speed and accuracy of response (introduction of breaks into lengthy/effortful activities such as examinations, motorway driving, assembly-line production). Also, variations in task or modality and the use of self- rather than system-paced schedules would be helpful. This would enable energetic demands to be distributed to alternate neural resources, and energy reserves to be re-established. Longer-term effects may manifest as reduction in regional brain volumes since brain areas with the highest energy demand will be most affected by a restricted energy supply and may be reduced in size. Novel forms of therapeutic agent and delivery system could be based on factors that regulate energy production and myelin synthesis. Since the phenomena and our proposed basis for it are not unique to ADHD but also manifests in other disorders, the implications of our hypotheses may be relevant to understanding and remediating these other conditions as well.

## 2. Background

Attention-Deficit/Hyperactivity Disorder (ADHD) is a highly heritable and heterogeneous condition with a widespread prevalence [1]. It often persists into adulthood with deleterious effects on educational, social, and occupational outcomes [2,3]. ADHD is defined by persisting, developmentally inappropriate, cross-situational, impairing levels of inattention, impulsiveness, and hyperactivity [4]. Our hypothesis focuses on a common observable feature of ADHD, marked moment-to-moment fluctuation in task performance [5-9]. Yet this ubiquitous phenomenon has been viewed as uninteresting random noise and ignored in the ADHD research field almost entirely until recently, when it was proposed as an aetiologically important characteristic requiring systematic analysis [10]. Behavioural and performance fluctuations are displayed over multiple time scales, but our primary interest here are with those that occur over seconds, rather than hours or days.

The genesis of this type of intra-individual performance variability (variability during continual responding to externally-paced stimuli) remains unknown. We propose that it arises from inefficient and inconsistent neuronal transmission of information, due to a deficient energy supply – lactate production – by the major *non-neuronal *component of the central nervous system (CNS), the astrocyte [11-13]. Astrocytes play a critical role in providing energy via lactate to rapidly firing neurons. Astrocytes can also provide lactate to oligodendrocytes, which is used as a substrate for myelin synthesis by oligodendrocytes [14], and thus enables rapid neurotransmission. The presence of receptors for the major brain neurotransmitters on astrocytes adds to the robust evidence for their direct involvement in neurotransmission [15-18]. Given that performance variability is not unique to ADHD but rather a common and unifying feature of several disorders, such as Traumatic Brain Injury, Schizophrenia, Narcolepsy, Phenylketonuria (PKU), as well as ADHD, we do not propose such variance as a specific marker of ADHD. Rather we argue that intra-individual response variability may be an important index of the efficiency of neural signalling which, in turn, is dependent on neurobiological regulation of brain function (e.g. factors that regulate the energy supply to neurons). Also, we suggest that it may account for a substantial proportion of the variance in performance of executive function tasks such that poor task performance may not reflect impaired executive function per se, but rather an admixture of poor neurobiological regulation of the external physiological environment of rapidly firing neurons as well as slowed processing speed arising from inadequately myelinated neurons, particularly those involved in working memory [19].

First we summarize the evidence for the transient fluctuations in task performance of individuals with ADHD, and the inadequacy of current explanations to account for the short time scales involved. Our hypothesis, introduced initially by Todd and Botteron [13], is then elaborated with evidence for important elements of the theory. Finally we outline strategies for testing the hypothesis and discuss its theoretical and clinical significance.

### 2.1 Transient fluctuations in task performance

#### 2.1.1 Intra-individual behavioural variability

We propose that increased intra-individual behavioural variability in continual responses to rapid, externally-paced stimuli are related to other factors than those causing increased variability in free-operant, non-externally paced tasks [8,9,20]. The former is timed by objective clock time, the latter by behavioural events not necessarily highly correlated with objective time. We define intra-individual variability as short-term fluctuations in the performance of an individual over a time-scale of seconds. It is differentiated from systematic and longer-lasting time-related changes in task performance due to practice, learning, developmental growth, or to remission or progression of a clinical condition or disease. This intra-individual variability will be the cause of inter-task variability. While not central in ADHD research, intra-individual response variability has been used as a primary dependent variable in research in normal ageing and in neurological populations [21-27]. Evidence that individuals with mild dementia exhibit greater intra-individual variability than both healthy adults and those with arthritis suggests that it reflects neurological compromise rather than deterioration of general health [22,28]. Moreover, findings from studies incorporating structural or functional human brain mapping methods indicate that intra-individual variability is not simply a sequela of general brain dysfunction, but is likely related to the functioning of neural circuits that engage the prefrontal cortex, particularly the dorsolateral areas [25,29,30]. Behavioural findings indicate that intra-individual response variability is a strong predictor of success [30], suggesting that poor performance on control tasks like Go-No-Go, inhibition versions of continuous performance tests (CPT), and stop-signal task may reflect problems in response variability rather than poor inhibitory control per se.

Prior to summarizing the empirical research on intra-individual variability in ADHD, a comment is warranted on the various approaches to its measurement. The most common method of characterizing variability in response or reaction times (both denoted here by RT) consists of a single-point estimate of the standard deviation (SD) around the mean reaction time (MRT) for each individual (ISD). This method has the advantage of simplicity, but because it aggregates response indices across time intervals, it conflates effects due to practice, learning, overall speed of responding, and randomness, with possible systematic periodic fluctuations in response times. Most importantly, response variability is usually strongly correlated with MRT [31]; computation of an intra-individual coefficient of variation (ICV = ISD/MRT) controls to some extent for the individual's overall speed of response. Two other measures capitalize to some extent on information that may be derived from serial analysis of response, but do not permit examination of potential periodicities within the moment-to-moment fluctuations in performance. Consecutive variability (CONV = √(∑(RTi - RT_i-1_)^2^/(*n *- 1)), where I = trial number, *n *= number of trials, √ = square root) provides a measure of local unpredictability (trial-to-trial variability) as well as controlling overall MRT. Autocorrelations (correlations between consecutive or subsequent observations) measure the extent of linear association between one response and subsequent responding, thereby providing insights into the consistency and predictability of behaviour [8,25]. Another approach has been to fit trial-by-trial RT data to the ex-Gaussian distribution, which provides parameters that may be related to more theoretically based distributions, such as the Wald [32]. This method permits determination of whether the increase in intra-individual RT variability in ADHD is a general phenomenon occurring across the whole RT distribution, or reflects a specific variability-producing process, such as attentional lapses, restricted to the tail at the slow end of the distribution [27]. The ex-Gaussian analysis delivers two measures of variability: one from the fast, Gaussian portion of the distribution, and one from the slow exponential tail [31]. In normal development and ageing, age-related change in intra-individual variability throughout childhood affects the distribution as a whole, whereas in adulthood, differences in variability appears to be due to factors influencing primarily the slow end of the RT distribution, such as attentional lapses [22,33].

The fast Fourier transform, which measures the power of periodic changes at different temporal frequencies, permits an assessment of periodicity over time [34]. Any periodically recurring patterns of responding within the RT series are manifest as peaks of power at particular frequencies.

#### 2.1.2 Intra-individual variability in ADHD

Significant and reliable differences in the speed and variability of responses have been documented between ADHD and comparison groups across a wide variety of neuropsychological tasks. Increased variability is seen in tasks requiring continual responses to rapid, externally-paced stimuli as well as in "free-operant" tasks without such a requirement. Recent studies of South African children from 7 ethnic groups and Norwegian children, using a computerized game-like free-operant task [8,9], showed significantly lower predictability of responding in ADHD than in non-ADHD groups. Predictability of response location and timing were measured in terms of variance explained by autocorrelations. Interestingly, in the free-operant task without externally-paced stimuli, response location – but not response timing – was a sensitive behavioural measure. In tasks requiring continual responses to rapid, externally-paced stimuli, however, children with ADHD display greater intra-individual variability in response times and are often found to respond more slowly and less accurately than their typically developing peers [6]. These performance differences have been found on the stop-signal task [35-38], a variety of CPT [39-41], time perception, time reproduction and motor timing tests [42-45], and visuomotor preparation tests [46]. Notably, a recent psychometric analysis of several measures of intra-individual variability (ISD, ICV, CONV) as well as measures of central tendency and shape of the reaction time distribution derived from children's performance of four different neuropsychological tasks (stop signal task, CPT, Go-No-Go, *n*-back working memory task) yield two important findings [47]. First, measures of intra-individual variability best discriminated between the ADHD and control groups; second, intra-individual variability appeared to be a unitary construct in ADHD (individuals with high variability on one task tended to have high variability on other tasks).

Poorer performance on neuropsychological tasks in ADHD is typically interpreted as evidence of executive function deficits, an interpretation that is based on slower and less accurate responses averaged across time. Accompanying group differences in intra-individual response variability are typically ignored – or, worse, become a nuisance parameter that keeps the inferential statistics from achieving significance. For example, the interpretation "poor inhibitory control" is commonly based on slower RTs to the stop signal or more commission errors in the CPT. Yet, it is often intra-individual variability in RT that correlates with the behavioural symptoms of ADHD, not the mean of the criterion variable [38,40,48,49]. Response variability correlates more strongly and reliably with ratings of ADHD symptoms compared to commission errors or other outcome measures on the CPT in a large epidemiological sample [40] and compared with stop signal reaction time (i.e. index of inhibitory control) in a large twin sample [38] or average response speed in a community sample of typically developing children [48].

Application of the ex-Gaussian model to RT data indicates that the greater response time variability in individuals with ADHD is most evident in the slow tail [50-52]: There appears to be some variability-producing process, such as momentary attentional lapses, that affects the slow RTs [50]. Recent evidence from Fourier analysis found that children with ADHD showed increased variability at all parts of the RT spectrum [53], consistent with Williams et al[54] who showed that intra-individual response variability in the fast tail of the distribution is distinct from that found in the slow tail. However, there is significantly (50%) more intra-individual variability in reaction time of individuals with ADHD at a modal frequency of around 0.05 Hz, indicative of lapses of attention 2 to 4 times per minute [53].

Variability is increased at *all *parts of the distribution [53] which is consistent with Williams et al. [54] who showed that that intra-individual response variability in the fast tail of the distribution is distinct from that found in the slow tail.

Intra-individual variability in response time has been proposed as a possible endophenotype with the potential to index genetic vulnerability to ADHD [10,55]. Further support for this proposition comes from two molecular genetic studies that report associations between the 10-repeat risk allele within the dopamine transporter gene (DAT1) and response variability in ADHD [56,57]. The dopamine transporter is the main site of action of methylphenidate, which reduces intra-individual response variability in ADHD [35,53,58].

##### 2.1.2.1 A theoretical model

The above models provide statistics descriptive of the data. In order to better constrain our hypotheses by data, we also develop a model of the cascade of energetic resources that we posit in H1. That supply chain has many similarities to hydraulic flow through *n *reservoirs [59], with the decay into the last *n*th state being analogous to the repletion of glutamate in the vesicles. Our hypothesis H1 predicts that the decreased efficiency in energy transport and glutamate restaging, which we posit to be characteristic of ADHD, will cause slower repletion, and entail larger values for the time constants that measure the mean occupancy times at each energetic stage. To simplify the model in light of available data, we assume equal time-constants for each stage. This model predicts a gamma distribution of the time required for the energy level at the last stage to reach the threshold necessary to support a response.

**Figure 1:**
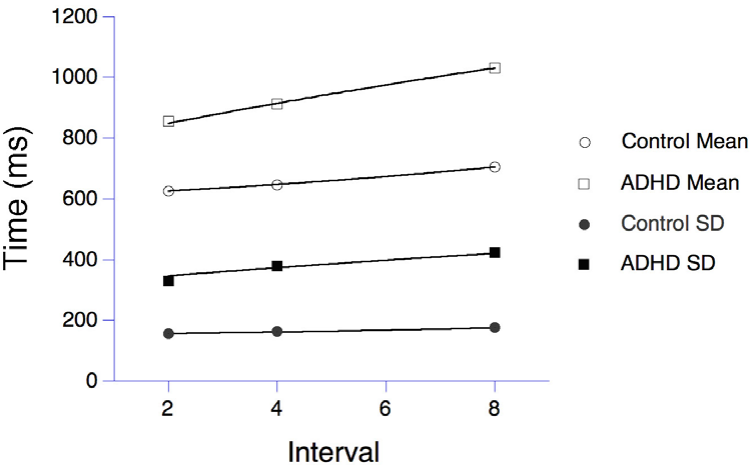
Means (open symbols) and standard deviations (filled symbols) of reaction times for a group of boys with ADHD (squares, *n *= 17) and age-matched control subjects (circles, *n *= 18) in a 4-choice reaction time task. The data are from Leth-Steensen et al. [52]. The lines through the data derive from a series latency mechanism described in the text.

The model has two parameters: the number of candidate stages (*n*), and their average time constants (τ). The gamma resembles the familiar ex-Gaussian distribution, and provides a good description of reaction-time data. Leth-Steensen, Elbaz and Douglas [52] compared the performance of 17 ADHD boys off medication, with a mean age of 11 years, with 18 age-matched control boys and ten 7 year-old boys, on a four-choice reaction-time task. Four warning circles were presented on a screen for 2, 4, or 8 s fore periods, followed by a change in the colour of one to yellow. The boys were to press a key corresponding to the changed stimulus. The authors fit ex-Gaussian densities to the observed distributions, and also reported the mean (μ) and variance (σ^2^) of the RT distributions. From those, the parameters of the gamma model may be deduced: *n *= μ^2^/σ^2 ^and τ = σ^2^/μ. This analysis imputed *n *= 6 stages for ADHD, and *n *= 16 for controls. The model cannot identify precisely which energetic processes correspond to these stages, but does indicate that there is a greater depth of resources available for the control group. As predicted, the processes are slower for ADHD, with time constants τ = 141, 152, and 171 ms for the 2 s, 4 s, and 8 s intervals, compared with τ = 39, 40, and 44 ms for controls. Holding parameters at these stipulated values, the gamma model recovered the statistics describing the data, as shown in Figure 1. The young control boys had intermediate parameters (*n *= 10 and τ = 40, 117, and 120 ms; their data are omitted from Figure 1 to emphasize the key comparison). The index of skewness of the gamma depends only on the number of stages, being 2*n*^-1/2 ^= 0.50 for controls and 0.82 for ADHD. This substantial increase in skewness is consistent with the differential increase in long response times for ADHD, and causes the larger variance seen in reaction times for this condition [60]. According to our model, the increase is explained by the slower replenishment in a cascade of energy reservoirs in ADHD individuals compared to controls.

**Figure 2:**
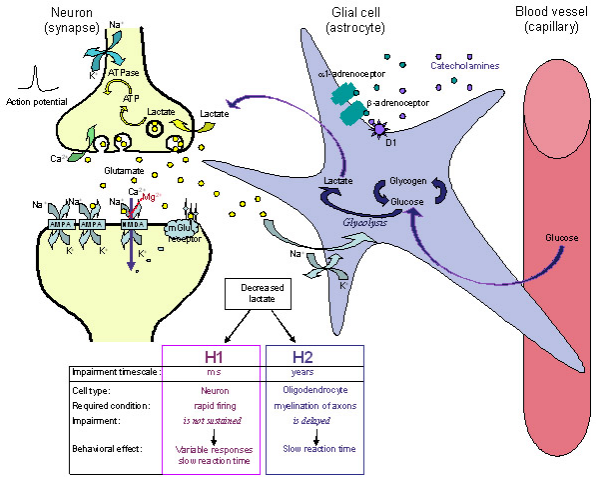
A scheme illustrating a glutamatergic neuron (left) a glial cell (astrocyte) and a small blood vessel (right) and the major components contributing to hypotheses 1 and 2 (H1 and H2). Neural activity triggers release of the neurotransmitter glutamate that is taken up into the astrocyte (via GLAST and GLT-1 transporters), and stimulates the breakdown of glycogen, the uptake of glucose, and glycolysis, to produce lactate. Rapid neuronal firing is sustained by the energy provided by the astrocyte-neuron lactate shuttle. Energy demands are high during rapid (burst) and maintained rates of neuronal firing. **H1: **At times of increased neuronal demand, deficient lactate results in decreased neuronal conversion of lactate to acetyl CoA, decreased ATP formation, deficient ATPase function, delayed restoration of ion gradients, elevated extracellular K^+^, deficient Na^+^-dependent transport of glutamate into astrocytes that is required to drive glycolysis and lactate release by the astrocytes. The result is that situationally appropriate firing rates are achieved only episodically. Methylphenidate treatment results in an increase of the extracellular levels of the catecholamines, NA (and DA) that stimulate glycolysis and release of lactate from the astrocytes. This is followed by glycogen replenishment, thereby correcting the energy deficiency, and restoring appropriate firing rates. **H2: **A deficient supply of lactate for oligodendrocytes in the developing nervous system slows and reduces the synthesis of fatty acids required for the synthesis of myelin. Poorly myelinated axons would transmit action potentials more slowly, accounting for inefficient integration (coherence) between brain regions and for slow reaction times. A number of neurotransmitter receptors present on astrocytes are not illustrated (e.g. muscarinic, α_2_, DA D3, D4, D5 and receptors for several neuropeptides).

The model is simplistic in that it assumes a common period (τ) for energetic processes that operate on multiple time-scales, with some processes in parallel, as shown in Figure 2. Comparison with other models, such as extreme-value distributions which also could tell a revealing story about the latent processes, awaits adequate data. More informative trial-by-trial analyses (e.g., [53]) will yield the fractal patterns of latencies first described by Hurst for analogous hydraulic systems [61], and found in reaction time distributions of normal subjects by Gilden [62]. It will require such detailed analyses to further resolve our hypothetical map between energy reservoirs and performance.

#### 2.1.3 Fluctuations in attentional processing and performance

Transient fluctuations have also been revealed in recent studies using innovative methods to capture shifts in attentional processing within relatively short time-scales, ranging from milliseconds to minutes. For example, rapid serial visual presentation (RSVP) paradigms such as the attentional blink design permit evaluation of temporal characteristics of information processing within a time-scale of milliseconds [63,64]. In this paradigm a stream of stimuli is presented briefly (100 ms) in the same location and in rapid succession (e.g., 10/s) and subjects are required to detect two targets in the stream. When the targets are presented within about 500 ms of one another, detection of the second target is impaired – a phenomenon termed the "attentional blink". Children and adults with ADHD and highly impulsive adolescents have been found to show a larger and more protracted attentional blink than their non-ADHD peers, suggesting less efficient attentional processing and/or more rapid depletion of processing resources [64-66].

Transient fluctuations in attentional state over a somewhat longer time-scale (30 s periods) have been demonstrated by means of error analysis on computerized CPT coupled with an infra-red motion analysis system. Analysis of attentional states over a period of 30-s revealed that children with ADHD exhibit many more shifts in attention states and spend much less time in an on-task attention state than their peers [67]. Fidgetiness, as detected by the motion-analysis system, was found to be related to the percent of time spent in the 'distracted' attentional state, in which the children's awareness of the relevant stimuli was diminished. These measures of attentional state provided more robust indicators of the performance differences between children with ADHD and comparison children than did the traditional time-averaged CPT summary measures of error rates, latency and variability [67]. Fidgetiness, that most public characteristic of ADHD, might be a by-product of fatigue of relevant neuronal circuits, and a search for new ones to engage.

#### 2.1.4 Drug effects on intra-individual variability and attentional fluctuations

Reaction time variability is reduced with appropriate monetary reward [45], but such rewards are not differentially effective for children with ADHD. The reason for this may be that reinforcers may be less effective for tasks requiring continual responses to rapid, externally-paced stimuli than for subject-paced (free-operant) responding [68]. The reason for this is probably that reinforcers work on free-operant behaviour, but not on instructed behaviour typical in tasks requiring continual responses to rapid, externally-paced stimuli (cf., [68]). Methylphenidate medication improves accuracy, speeds reaction time, and reduces reaction time variability in individuals with ADHD [35,58]. Moreover, methylphenidate has been shown to improve time on-task, with concomitant decreases in the number of shifts in attention as the task progressed [67]. Such treatment effects suggest that the increased availability of extra-neuronal catecholamines arising from the blockade of their uptake by methylphenidate could lead to increased activity of noradrenaline at β-adrenoceptors and possibly also dopamine acting at D1 receptors found on astrocytes [15,69]. A key fact is that stimulation of α_1_- and β-adrenoceptors on astrocytes enhances glycolysis and lactate production [69]. Dopamine acting on D1 receptors may have similar effects, but these are not well documented.

#### 2.1.5 Existing explanations and their limitations

At present, various tasks used to measure different aspects of executive function usually have moderately heavy cognitive demands and share requirements for continuous speeded responding to computer- or experimenter-produced stimuli, rather than being self-paced. Experimenter-paced measures of working memory are substantially better predictors of reading letter span, continuous operation span, literacy and mathematics scores than are more traditional, subject-paced measures [70]. We believe they provide better diagnostics of ADHD because, like stress tests for cardiac function, they most directly challenge the subjects' short-term reserves of energy, and that is a major resource compromised in ADHD.

Slowing of MRT and increasing RT variability over time has been postulated to reflect mental fatigue, 'resource depletion', motivational deficits, fluctuations in executive control, or underlying neurobiological disturbances (e.g., motor timing deficits), which are caused by the need for continuous speeded responding to high-demand tasks [6,20,71-75]. Most of these models cannot account for the moment-to-moment fluctuations in response accuracy characteristic of ADHD.

The dynamic developmental theory of ADHD [8,9,20] explains intra-individual behavioural variability as deficient acquisition of stimulus control of long chains of behaviour. This deficiency is rooted in reduced efficacy of reinforcers ("rewards") combined with poorer extinction ("unlearning") of inefficient behaviour. The dynamic developmental theory addresses free-operant behaviour without time constraints. However, neither the dynamic developmental theory of ADHD, nor other theories and explanations account for the intra-individual performance variability of ADHD on tasks that require continual responses to rapid, externally-paced stimuli. The present paper addresses this aspect by postulating that reaction time variability and other manifestations of transient fluctuations in performance, which arise in the context of the need for continuous rapid neuronal firing, reflect the effects of transient depletion of neuronal energy on the efficiency of information processing.

## 3. The hypothesis

In this section we highlight the strengths and limitations of Todd and Botteron's Energy-Deficiency Model (section 3.1), present a brief background on astrocytes and their role in the development and function of the CNS (section 3.2), formulate the hypotheses (H1 and H2: section 3.3), and provide a detailed summary of evidence supporting the hypotheses (section 3.4).

### 3.1 Energy-deficiency model

According to Todd and Botteron [13], the genesis of the behavioural symptoms of ADHD is linked directly to impairments of the astrocyte-neuron lactate/energy-shuttle that is based on the astrocytic uptake of glucose from blood capillaries, its utilization (conversion to lactate) and storage as glycogen [76]. Since acute amphetamine treatment of young adults has been reported to stimulate glucose uptake in the frontal lobes [77], Todd and Botteron hypothesized that reduced catecholaminergic input (in ADHD) leads to a decrease in astrocyte-mediated neuronal energy metabolism and impaired frontal function.

Todd and Botteron's model links catecholaminergic activity to the regulation of neuronal energy metabolism. But it does not make explicit how a decreased neuronal energy supply mediated by hypofunctioning catecholamines might alter either cognitive performance based on frontal lobe function or account for ADHD symptomatology in general. In contrast, our hypothesis addresses a specific aspect of the clinical presentation, – namely, moment-to-moment fluctuations in task performance that are often also manifest in general behaviour – and we refine and extend the biochemical bases that underlie this hypothesis.

### 3.2 Astrocyte and oligodendrocyte function in the central nervous system (CNS)

The CNS comprises two main types of cells: neurons that are directly involved in information processing, and glial cells (astrocytes, oligodendrocytes and microglia) which play a major role in the development and mature function of the CNS. Astrocytes are responsible for maintaining the environment of cells in the CNS: they provide nutrients and modulate the release and uptake of glutamate, electrolytes (Na^+^/K^+^) and other by-products of neural activity [78]. In addition, astrocytes play an important role in neural signalling. They have receptors for neurotransmitters such as glutamate, GABA, acetylcholine and the monoamines as well as for other neuromodulators including neuropeptides, cytokines and steroid hormones [15-18,79-84]. They can modulate the excitability of neurons via oscillations of Ca^2+ ^levels initiated by stimulation of metabotropic glutamate receptors [15,85-90].

Oligodendrocytes are responsible for forming myelin, a membranous sheath that is wound spirally around axons, providing electrical insulation that leads to a more than 10-fold increase in the speed of signal transmission occurring in unmyelinated axons [11].

### 3.3 Statement of the hypothesis

Lactate production by astrocytes is insufficient during brief periods of increased demand in ADHD. This hypothesis has two consequences (Figure 2). **H1) **The inability of astrocytes to provide an adequate supply of lactate to rapidly firing neurons results in a localized and transient deficiency in ATP production, impaired restoration of ionic gradients across neuronal membranes and slowed neuronal firing. This leads to inconsistent performance of demanding cognitive tasks; **H2) **Insufficient provision of lactate for oligodendrocyte function in the longer term gives rise to deficient fatty acid synthesis and delayed or reduced myelination of axons. In turn this leads to less efficient transmission and longer reaction times. The hypothesis refers to two effects over very different time scales: The effects on rapidly firing neurons occur over milliseconds (H1), whereas the effects of reduced lactate supply for oligodendrocytes when demands are high during development takes place over months and years (H2). Collectively the neural outcomes of astrocyte dysfunction would manifest behaviourally as inefficient and/or inconsistent performance (i.e. slow and/or variable response times across the lifespan, particularly during tasks that require continuous speeded responses and complex information processing).

### 3.4 The hypothesis – discussion

#### 3.4.1 H1: Impaired astrocyte function limits energy (lactate) supply to rapidly firing neurons

The ionic composition of the cell cytoplasm is very different from extracellular ion concentrations. In the neuron the ionic gradients across the membrane constitute a store of potential energy that can drive the influx of Na^+ ^and efflux of K^+ ^ions to generate an action potential, and the influx of Ca^2+ ^ions to trigger neurotransmitter release and to generate Ca^2+ ^waves in and between both neurons and glial cells. To maintain repeated firing over an extended time, neurons require energy to restore the trans-membrane gradients of Na^+^, K^+ ^and Ca^2+ ^ions. Neurons generate the necessary energy in the form of ATP; this drives the membrane-associated Na^+^/K^+^ATPase to pump Na^+ ^out of the cell and K^+ ^back into the cell. ATP is also required by Ca^2+^ATPase to pump Ca^2+ ^out of the cell or into intracellular stores (Figure 2).

Neuronal activity is tightly coupled to glucose utilization [91]. Neural activity triggers oxidative metabolism (to produce ATP) within neurons followed by breakdown of energy stores (glycogen in astrocytes) and uptake of glucose from blood capillaries into astrocytes to produce lactate [91]. Rapid neuronal firing, however, is sustained by the astrocyte-neuron lactate shuttle [92]. Lactate is the essential energy source for rapidly firing neurons. It is a more efficient fuel than glucose because it is metabolized to form ATP more rapidly and, unlike glucose, does not require ATP for its metabolism [76]. It is imperative for neurons to make use of the most efficient energy supplies when rapid neural processing is required and demands for energy are high, and the brain has evolved ways to do so [93]. The neurotransmitter glutamate (released by the majority of excitatory neurons in the CNS) stimulates glycolysis (glucose utilization and lactate production) in astrocytes and release of lactate into the extracellular fluid [91,94]. An inadequate supply of lactate during periods of rapid neuronal firing, when local energy demand is high, briefly impairs neuronal function, particularly during the latter part of a train of neural impulses and shortly thereafter, causing an extended refractory period. This increased latency to new information is measured in the attentional blink paradigm (section 2.1.3). Compromised energy supply, as hypothesized for ADHD, should impair response inhibition or alternation at these times.

Periods of rapid neuronal firing will be followed by slow unsynchronized firing exerting less demand on energy resources, allowing replenishment of energy reserves and restoration of function. Energy reserves will depend on the prior history of neural and astrocyte activity. Brief periods of energy insufficiency followed by periods of normal supply are proposed to account for the variability of behavioural response seen in ADHD when performing complex tasks that require speed and accuracy.

##### 3.4.1.1 Impaired maintenance of ion gradients across the neuronal membrane

Decreased availability of lactate to produce energy in the form of ATP would impair the function of membrane-associated Na^+^/K^+^ATPase and Ca^2+^ATPase pumps, resulting in elevated extracellular K^+ ^and decreased Ca^2+ ^and Na^+ ^concentrations. Consistent with this hypothesis, ADHD children were reported to have decreased urinary excretion of Ca^2+^, Na^+ ^and phosphate, but not K^+ ^ions [95]. Failure to maintain homeostasis of inorganic ions will alter electrochemical gradients across neuronal and glial cell membranes. As the resting membrane potential of neurons is dominated by the K^+ ^concentration gradient across the membrane, failure to reduce elevated extracellular K^+ ^concentrations following neural activity will alter membrane potentials, cause depolarization to last longer than required, and thus impair neuronal function. Raised K^+^/reduced Na^+ ^gradients are likely to have widespread effects in different parts of the brain, promoting for example monoamine release (e.g. dopamine [96]) and altering the conformation of transporters, thus affecting monoamine uptake (e.g. dopamine [97] 5-HT [98]).

##### 3.4.1.2 Impaired uptake and removal of extracellular glutamate

Astrocytes normally maintain low extracellular levels of glutamate and K^+ ^[78]. Glutamate transport into astrocytes is driven by the influx of Na^+ ^along its electrochemical gradient. But the low membrane potential resulting from the astrocytes being less able to maintain low extracellular K^+ ^concentrations in the ADHD condition will diminish the electromotive drive for Na^+ ^influx and thereby hamper removal of glutamate from the extracellular fluid [93,99,100]. Failure to maintain low extracellular glutamate levels will impair glutamate neurotransmitter function, neuroplasticity, learning and memory, and could lead to excitotoxicity and cell death, reflected as reduced CNS gray matter. (Small reductions in grey matter are reported for subjects with ADHD [101-103]).

##### 3.4.1.3 Impaired neuromodulator regulation of lactate formation

Several neurotransmitters can potentially modulate lactate production in astrocytes, with the majority of evidence supporting a role for noradrenaline acting on α_1_-, α_2_- and β-adrenoceptors [88,104-106]. This is important in view of widely accepted explanations of ADHD symptoms in terms of catecholamine function [107], psychostimulant effects on glucose utilization (section 3.1) and efficacy as medication [108]. Glial receptors for dopamine (D1–5) [15,88] and serotonin (5-HT2) [109] have also been reported but their effects on lactate production in astrocytes have not been well documented.

Extracellular lactate decreases immediately after neuronal activation, but rises again after a short delay [110]. Normally within milliseconds of glial β- or α_2_-adrenoceptor activation noradrenaline induces the breakdown of glycogen to glucose (glycogenolysis) to supply the needed lactate [69,105,111,112]. This is followed by a phase of glycogen re-synthesis in the astrocytes that can last several hours [112]. Failure to adequately replenish glycogen stores in the astrocytes would reduce the availability of energy substrates required for subsequent or sustained neuronal activity. Therapeutic agents (e.g. methylphenidate, amphetamine, atomoxetine, desipramine, modafinil) block the noradrenaline transporter and increase extracellular concentrations of noradrenaline which stimulates glycolysis and lactate production in astrocytes. Although evidence strongly supports a role for noradrenaline, both dopamine and serotonin are known to modulate cAMP levels in astrocytes and could therefore also play a role [15,109].

Glucocorticoid hormones also modulate the supply of energy [113,114]. Glucocorticoids inhibit glucose transport into neurons and astrocytes and inhibit glycogen synthesis stimulated by noradrenergic activity [115]. Thus, glial activity may be down-regulated by increased hypothalamo-pituitary-adrenal activity in situations perceived as stressful (e.g. expressed emotion in the family of ADHD children or the challenge provided by cognitive tasks presented to the children). High levels of glucocorticoids acting on astrocytes would impair glycogen replenishment, deplete energy reserves, and reduce lactate transport. Behaviourally, this will give rise to fatigue and predispose to psychological problems, as has been proposed for other disorders such as Parkinsonism and major depression [116].

Cytokines, small proteins that support communication between cells of the immune system, can be produced by and influence the function of astrocytes (e.g. TNF-alpha, IL-1, IL-6 [117]). Reduced stimulation of β-adrenoceptors leads not only to decreased glycogenolysis but also to impaired production of several growth factors (e.g. nerve growth factor (NGF), basic fibroblast growth factor (basic FGF), transforming growth factor-beta1 (TGF-beta1) along with increased production of nitric oxide and the pro-inflammatory cytokines [118,119]. Cytokine exposure can lead to an overproduction of nitric oxide and its metabolites that diffuse out and damage mitochondria and the energy supply in nearby cells (including neurons [120]). TNF-alpha and IL-1 can fundamentally perturb the energy metabolism of astrocytes promoting the uptake of glucose without either storing it as glycogen or releasing lactate [121]. This disruption can therefore not only impair short-term demands for energy, but also the long-term requirements for development (see hypothesis H2, section 3.4.3), that in the worst case can lead to apoptosis of the oligodendrocyte [118]. Other cytokines (e.g. the calcium-binding S100B) also regulate energy metabolism, promote neuronal survival and regeneration [122]. Dopamine also stimulates release of growth factors (including NGF, glial-derived nerve growth factor, GDNF and brain-derived nerve growth factor, BDNF) and therefore plays a crucial role in development of the brain and maturation of the nervous system [84].

#### 3.4.2 Preliminary evidence: impaired energy supply during rapid neuronal firing in ADHD

This section first considers the limited direct neuroimaging evidence of altered energy utilization in the CNS of people with ADHD that could potentially derive from deficient astrocyte function [116]. We then discuss electroencephalographic (EEG) recordings indicating inefficient communication within and between neuron clusters that could be attributed to impaired energy supplies.

Energy utilization in the brain associated with the cognitive challenge of CPT performance was measured by positron emission tomography (PET) and deoxyglucose in 25 adults with childhood onset of ADHD [123]. The authors reported decreases in utilization in 30 of 60 regions analysed, and a global reduction of >8%. The largest decreases were registered in adjacent regions of the superior frontal, premotor, and somatosensory cortices. Methylphenidate in healthy volunteers has been shown to increase glucose utilization in these and other brain regions (e.g. cerebellum), perhaps by elevating extracellular noradrenaline concentrations. Decreased glucose utilization in ADHD may result from inadequate noradrenergic stimulation of astrocytes. But decreased glucose utilization in striatum and differences following acute *vs*. chronic methylphenidate treatment are results that warn against making simple generalizations about the effects of stimulants on energy utilization [124].

Some EEG measures indicative of ADHD may reflect impaired immediate energy supply from astrocytes (H-1) and others impaired myelination (H-2, section 3.4.3/4) deriving from a longer-term lactate deficiency. In either case the presence or absence of effort to overcome the inefficiency should be visible in terms of the amplitude of event-related potential (ERP) components in the EEG representing the stages of information processing, and deficient levels of 'activation' in the ERP latencies.

The contingent negative variation (CNV) is an excitatory, slow, negative-going waveform that occurs in the second before a stimulus. The CNV is usually considered to index anticipatory and preparatory processes to a stimulus that may require a response. It has been reported to be reduced in amplitude in people with ADHD [125-127], although this was not replicated in two further reports [128,129]. The extent to which the reduction of CNV reflects reduced effort in or motivation for preparation is secondary to the point at issue here, namely that subjects with ADHD are poorly prepared for the stimulus. This may be due to impaired learning, or poor association of actions with delayed outcomes in previous experiences, as suggested by Sagvolden et al. [20], and this is non-adaptive to the demands of the situation. The P3 component elicited by a rare or meaningful stimulus represents updating of working memory-like templates of its associations. The amplitude is reduced in most studies of ADHD. It has been argued that reduced effort makes a contribution to this effect [130,131]. These are two of several ERP markers reflecting inefficient information processing in ADHD. We propose that insufficient energy reserves underlie this deficiency.

Variable ERP latencies are widely reported, especially for the P3. This variability is evident in 8–9 year-old children born very prematurely and given the diagnosis of ADHD, compared to those who had a similarly low birth weight without the ADHD diagnosis [132]. Methylphenidate treatment decreased variability both of the P3 component [133,134] and the response times recorded [135,136]. The number of responses that were too early (premature, impulsive) or too late were reduced with psychostimulant treatment [135,137]. Effective therapeutic drugs blocked dopamine and noradrenaline transporters and increased extracellular levels of catecholamines permitting activation of β-adrenoceptors (and/or D1 receptors) on astrocytes to stimulate lactate production.

#### 3.4.3 H2. Impaired oligodendrocyte function impairs axon myelination

In the human brain the myelination of axons – a principle function of the oligodendrocytes – starts *in utero *and continues through the first 3–4 decades of life [138]. The increase of white matter during development puts high demands on metabolism. Myelin starts to form before birth with much being laid down in the first two years of life. Myelination is prominent in the prefrontal cortex, and of course in the major fibre tracts, especially the corpus callosum – with levels of "adult functionality" achieved normally during adolescence [139,140]. Impaired myelin synthesis as a result of intermittently insufficient lactate production by astrocytes would have a detrimental effect on the coordinated myelination of axons during development, and lead to impaired communication between brain regions and poor integration of information in these target brain structures.

Oligodendrocytes normally oxidise glucose and lactate at far higher rates than either neurons or astrocytes [14,141]. Glucose is metabolised in the pentose phosphate pathway to produce NADPH and in the glycolytic pathway to produce lactate, both of which are used to synthesize lipids: lactate is the preferred substrate for fatty acid synthesis and myelin formation. As oligodendrocytes are the highest lactate and glucose consumers in the developing brain, any deficiency in the supply of lactate would impair lipid synthesis and retard myelination of axons. Impaired myelination reduces the speed of conduction of action potentials in individual neurons and could account for slow reaction times. Axons that are not properly insulated by the myelin sheath would be less efficient at conducting action potentials and require more energy resources than normal. Partial myelination of fibre pathways would also contribute to impaired integration of information in target regions and provide a further source of response variability.

Myelination is a sensitive indicator of functional brain maturation. Across at least the first two decades of development myelination consists of a broad increase in overall white matter density as well as a more region-specific progression, proceeding from posterior to more anterior regions, [142-145]. In magnetic resonance spectroscopy (MRS) and diffusion tensor imaging (DTI) studies, maturation of relatively restricted regions of white matter has been found to correlate with the development of cognitive functions such as working memory capacity (left frontal regions), reading ability (left temporal lobe) and even with IQ (bilateral association areas [144,146]. In a study of 100 children with evidence of delayed development but otherwise normal MR-scans, Pujol and colleagues [143] were able to show a reduction of myelination (in part asymmetric) equivalent to normally developing children who were some 3 years younger. Also, at the other end of the lifespan, white matter damage due to axonal loss that occurs in normal ageing has been found to correlate with working memory performance, even after controlling for age [19]. This suggests that working memory performance may be particularly dependent on complex networks, which in turn depend upon white matter connections. These studies conclude that there is a positive relationship between the density and organization of myelinated fibres and the efficiency (maturity) of cognitive function [144].

#### 3.4.4 Preliminary evidence of altered myelination in ADHD

##### 3.4.4.1 Neuroimaging

Evidence for impaired and/or delayed myelination derives from MR measures of white matter density and the integrity of myelinated neuronal pathways indexed by DTI and MRS measures of metabolites (e.g. N-acetyl-aspartate, NAA). Support for the functional impairment of these pathways comes from EEG recordings (section 3.4.4.2).

Seven of the 8 anatomical MRI studies report decreased total white matter volumes in children and adolescents with ADHD [102,103,147-150]. In the largest MRI study that scanned over 150 boys with ADHD on at least two separate occasions, the reduction in white matter volume was substantial (a 10% reduction) compared to those who had been treated with stimulant medication or had no diagnosis [103]. Indeed, one study linked smaller white matter volumes with slower processing speed, as indexed by the speed of colour-naming [150]. These results implicate a contribution of delayed myelination to ADHD cognition.

DTI provides a measure (fractional anisotropy, FA) of the coherence and integrity of the biochemical microstructure of myelinated pathways. An initial study comparing a group of 18 children with ADHD with 15 matched controls recorded a reduced FA in the right cerebral peduncle/anterior limb of the internal capsule (right neostriatum and premotor cortex), and in the left middle cerebellar peduncle (left cerebellum and parieto-occipital region). Ashtari and colleagues report that the lower the cerebellar FA, the more severe were the ratings of symptoms of inattention [151]. These first findings point to a link between white matter anomalies and the symptomatology of ADHD.

Such findings are extended by ^1^H-MRS measures of neuronal metabolism in the brains of subjects with ADHD. With this method creatine, choline, taurine, inositol, NAA, glutamate and lactate can be detected and measured. Unfortunately there have been scant attempts to report on the last two. As yet creatine, choline (and their phospho-derivatives) – regarded as indicators of lipid metabolism and membrane integrity – have also not been studied in ADHD. The easiest metabolite to measure is NAA. NAA is found in neurons, not in glia, and is regarded as a marker of neuronal density, function, viability and perhaps functional connectivity [152]. It is synthesised by the enzyme NAA transferase in neuronal mitochondria from acetyl coenzyme A (acetyl Co-A) and aspartate, and used by oligodendrocytes to produce acetyl groups for the synthesis of myelin lipids [153,154]. During development NAA levels increase (as choline levels fall) and reflect increased synthesis (or decreased utilization) in the formation of myelin [155,156]. High NAA concentrations correlate with increased ADP [157,158] as acetyl CoA levels required for ATP synthesis are depleted. Decreases of NAA levels reflecting neuronal dysfunction are associated with neuronal loss in certain parts of the brain in many major psychiatric illnesses [159].

Increased NAA levels are reported for the right frontal lobe [160] and fibres entering/leaving left frontal regions (centrum semi-ovale [161]) of ADHD subjects compared to healthy and autistic comparison groups. But group differences were not found in 2 studies of the right frontal lobe [162,163] and decreases were reported in small studies for the left frontal [164] and right lenticular regions [165]. However Yeo et al. [162] noted that the smaller right frontal volume for their 17 ADHD children correlated with NAA and choline measures, and that NAA levels in turn related to performance on a sustained attention task. Elsewhere, intriguingly, raised frontal glutamate concentrations were noted, particularly on the right [160,163]. As a cautionary note, it should be pointed out that animal work has shown that methylphenidate treatment can lead to increased cortical levels of NAA [166]. Further, the results of these 6 ADHD studies must be regarded as preliminary as they each represent very small subject groups, and the range of brain regions they could sample was very restricted. Nonetheless it should be noted that all the studies recorded changes, and unusually some subjects show raised metabolite levels (NAA, choline, glutamate). These results point to changed patterns and rates of myelin synthesis (and breakdown) that may reflect intermittently insufficient supplies of lactate. The result is an overall decrease of white matter for a given age-class, as described above.

##### 3.4.4.2 Neurophysiology

We propose that the functional consequences of the impaired/delayed developmental laying down of the myelin sheath can be seen in three types of EEG measure: evoked potential (EP) latencies, the topographic distribution of the power spectrum in the quantitative EEG, and in the coherence of the EEG waveforms between brain regions.

EPs representing sensory information ascending in the auditory nerve [167] and the brain stem appear at longer than normal latencies (e.g., components III and V). Indeed, the transmission times from components I to III and I to V are reported to be increased in subjects with ADHD [168]. The latency of the steady state visual EP in the frontal cortex of ADHD children is markedly delayed [169]. Indeed, in their report of a delayed velocity index for EPs in ADHD, Ucles et al [170] proposed not only that abnormal myelination in the cortico-spinal path could be responsible, but that the result could be indicative of a much more widespread problem.

A large proportion of patients with ADHD (or narcolepsy, see 3.5.1 below) demonstrate an increased ratio of relative theta to alpha or beta power in the EEG, especially over anterior brain regions [171,172]. One explanation of the dominant lower firing frequencies could lie with the reduced lactate availability required to sustain rapidly firing neurons. There is usually a marked normalization of this balance between oscillation frequencies after methylphenidate treatment [173]. In the unmedicated sample there is a positive correlation between P2, N2 and P3 ERP latencies, widely reported to be delayed [137] and increased theta power [174,175]. A plausible reason for this shift in balance between oscillation frequencies lies in a decreased representation of the faster frequencies owing to deficient neuronal energy supply (H1) and/or reduced myelination in brain stem reticular sources active in generating some of these rhythms [170] which is consistent with our hypothesis H2.

A more direct measure of the coupling of activity between brain regions can be estimated by EEG coherence of waveform between recording sites. Coherence can be conceptualised as the correlation in the time domain between 2 signals in a given frequency band. EEG coherence normally develops systematically with age in a non-linear fashion. There is evidence of development in longer-range inter-hemispheric coherences which are not apparent in boys with ADHD. Furthermore, boys with ADHD show elevated slow-wave coherences and reduced fast-wave coherences between hemispheres, although within hemispheres the coherence in the theta band is reduced, especially over frontal regions [176-178]. This is most easily explained by unusual if not delayed development of the large white matter tracts connecting brain regions. At short distances between signals the increased coherence at slow wave frequencies in children with ADHD is viewed as consistent with a delay in the pruning back of over-produced synapses and local connections [179]. As would be expected such long-term alterations remain unaffected by short-term methylphenidate treatment [180].

### 3.5 Supportive evidence from related disorders

We propose that a disturbed lactate shuttle in ADHD accounts for brief transient impairments in rapid neuronal firing and delayed myelination: Together they result in variable responses. These impairments may account for similar phenomena in other neurodevelopmental disorders. We do not regard our hypothesis as specific to ADHD – for example, individuals with schizophrenia have been found to respond more slowly and variably on attentional and cognitive tasks [181,182] – nor do we suggest that it occurs in all psychiatric disorders. In the following sections we select two disorders, PKU and narcolepsy, to illustrate the presence of phenomena that appear to model the situation that pertains to ADHD.

#### 3.5.1 Parallel pathological conditions: Phenylketonuria (PKU)

PKU results from high concentrations of phenylalanine (Phe), that arise from an inability to convert it into tyrosine, and which inhibit transport across the blood brain barrier of neutral amino acids such as tyrosine and tryptophan necessary for the synthesis of the three principle monoamine transmitters. If children are placed on Phe-free diet early enough, microcephaly, mental retardation and motor problems can be avoided, although sub-clinical symptoms may remain, particularly in the cognitive domain [183,184]. Children with PKU (off-diet) are rated by parents and teachers as more distractible, hyperactive, and impulsive than healthy controls [185], with many symptoms similar to ADHD (e.g. restlessness, fidgeting, concentration difficulty, short attention span, low frustration tolerance [186]). Realmuto et al. [187] noted that 9/13 of their subjects either manifested or had a history of comorbidity with ADHD. Prenatal exposure was associated with a higher likelihood of expressing hyperactive/impulsive symptoms and postnatal exposure was associated with a higher likelihood of expressing inattentive symptoms [188]. Indeed, a considerable proportion of people with treated PKU take psychostimulant medication for their attentional problems (e.g. 26% of a sample of 38 school-aged children with PKU [189]).

Cognitive impairments of individuals with PKU like those with ADHD include variable reaction times, sustained attention, working memory, executive function, planning, learning, tests of colour-naming, arithmetic, verbal abilities and academic performance [187,190-199]. Inter-hemispheric interactions, as measured by slowed transfer times [200], and a lack of the across-hemisphere advantage in performing a name-identity task [183], are affected in comparison to neurologically intact children. Neurophysiological studies report slow latencies for several early EPs [201], the later P3 component and slower more variable motor reaction times [194,195,202]. In the EEG slow (theta) rhythms are characteristic of individuals with PKU. Indeed their high theta/alpha ratio (recalling ADHD) was sensitive to treatment reducing the levels of Phe [203,204].

Functionally, these features reflect delayed myelination, low monoamine levels, and impaired energy availability. Impaired myelination is the primary neuropathological feature of treated or untreated people with PKU: A delay is typical of the younger ages and diffuse demyelination is reported at older ages [205-208]. The finding of low HVA levels (dopamine metabolite) in the CSF [209] confirms the second feature, low dopamine levels. Importantly, changes of dopamine in the rodent model go hand in hand with the depletion and restoration of Phe levels [210]. Noradrenaline and serotonin would also be expected to be affected. The third feature was addressed in 11 young adults with PKU using phosphate MRS. Pietz et al. [204] describe changes of cerebral energy metabolism that could underlie reduced transmission speed, myelination and catecholamine availability [205]. Among 11 measures taken at baseline, only ADP was significantly elevated, and inorganic phosphate decreased. Phe loading then decreased phosphocreatine and ATP levels while further increasing ADP. This is consistent with Phe inhibition of pyruvate kinase and the concurrent conversion of ADP to ATP [211]. Impaired pyruvate synthesis would reduce lactate production and the ability of astrocytes to meet the energy requirements of sustained rapid neural firing and a shortage of lactate would also impair the ability of oligodendrocytes to synthesize myelin.

#### 3.5.2 Parallel pathological conditions: narcolepsy

Narcolepsy is a neurological disorder characterized by excessive daytime sleepiness [212]. Some of the neuropsychological characteristics of narcolepsy are strikingly similar to those seen in ADHD and children with ADHD have an increased tendency to daytime sleepiness [213]. Individuals with narcolepsy have slower reaction times and more within-task variability of performance than control subjects on a variety of attentional tasks ranging from those sensitive to arousal and sustained attention, to the executive control of attention [214]. Recent studies report narcolepsy-related deficits in attentional and executive function which place high demands on inhibition and task management, but not on simple tasks of memory and attention [214,215]. The pattern of findings was thought to be indicative of a depletion of available cognitive processing resources because of the need for continuous allocation of resources to monitoring.

Like ADHD, hypoarousal (sleepiness) is induced in situations of low stimulation, such as reading or boring repetitive tasks [112,216-218]. Similarly, children with ADHD or narcolepsy appear to use motor overactivity and fidgetiness to counteract their drowsiness [218-220]. It is interesting to speculate how a deficiency in energy supply might contribute to the other symptoms of ADHD, such as hyperactivity. Depression of sensory modules could certainly lead to a suboptimal level of stimulation, inducing sensation-seeking behaviour, with motor, vocal, and other activities displacing the contextually "appropriate" behaviours, which through local energy deficiency can no longer provide the necessary arousal. Derangements of calcium-dependent protein phosphatase and kinase activity impair working memory [221]. These and other implications of the energetics hypothesis require further consideration, lying beyond the scope of this paper. Lastly, as in ADHD analyses, EEG rhythms show the ratio of theta to alpha or beta power in narcolepsy to be higher than normal [172]. Narcolepsy has been attributed to depletion of the transmitter hypocretin, a hypothalamic neuropeptide that regulates energy metabolism and dopamine activity in certain brain regions [222-224]. Modafinil, the drug normally used to treat narcolepsy, is effective in treating ADHD and can inhibit dopamine re-uptake [220,225-228]. It is also an α_1_-adrenoceptor agonist and can therefore facilitate β-adrenoceptor-stimulated lactate production in astrocytes [229,230]. Thus, the mechanism of treatment could be the same in both disorders. This highlights the similarities between the disorders that could reflect common underlying disturbances.

## 4. Testing the hypotheses

The fundamental tenet of our hypotheses (H-1 and H-2) – to be tested – is that a deficient energy supply to rapidly firing neurons (the lactate shuttle) underlies moment-to-moment fluctuations in response speed and accuracy (astrocyte mechanisms) and, in the long-term, episodes of lactate deficiency during development delays axon myelination (oligodendrocyte function). These two links need to be demonstrated (section 4.1).

The consequences of these disturbances are proposed to lie with a) decreased neural activity when sustained rapid firing is required (neurophysiological level), b) delayed and variable cohesion between the components of the neural circuitry responsible for integration of information and selection/organization of appropriate response (psychophysiology), and c) the poorly coordinated and intra-individual variability of performance typical of individuals with ADHD. More evidence is required (section 4.2).

### 4.1 Is there an energy deficiency?

There are several points in the energy cycle where dysfunction could occur. The present hypothesis focuses on one specific aspect: The provision of adequate amounts of the more efficient metabolic fuel, lactate (rather than glucose), to rapidly firing neurons (e.g. glutamate transporter, mitochondria, monoaminergic regulation of astrocyte function: section 4.1.1). Identification of environmental and/or genetic origins of the lactate deficiency would contribute to an understanding of intra-individual variability in performance of high energy-demanding tasks (section 4.1.2). Factors that impair myelination may contribute to slow responsiveness and are also reviewed (section 4.1.2).

#### 4.1.1. The performance of energetic compartments

There is a need for more precise measures of the dynamics within and between the major compartments of energy production, storage and utilization through studies using labelling, stimulation and inhibition of the various constituents in animal models of ADHD and tissue culture (e.g. delineation of the quantitative relationship between neuronal firing rate and lactate utilization, intra/extracellular glutamate flow, mitochondrial function (ATP/ADP ratio and oxygen consumption), effects of lactate restriction on ionic gradients, function of regulatory factors (e.g. neurotransmitter/neuropeptide receptors, particularly noradrenergic α_1_-, α_2_- and β-adrenoceptors), effect of impaired lactate production on myelin synthesis (and accumulation of NAA), influence of NAA availability on acetyl-group incorporation into myelin). Regional differences in measures of effects of transient energy deficiency are important to note, since the hypothesis does not predict that all parts of the brain will be affected equally. Greater effects (e.g. reduced size) should be observed in those brain areas that contain or form part of rapidly firing neural circuits that transiently deplete local energy reserves and hinder synapse formation.

Measures of some of these effects are becoming technically feasible for human studies, *in vivo*, with MRS. For example, changes in glutamate production (tricarboxylic acid cycle), lactate synthesis (glycolysis) and glutamine synthesis have been demonstrated in neurological patients using labelled carbon (^13^C) MRS [231,232]. Further data on the energy metabolites ATP, ADP, inorganic phosphate, phosphocreatine and creatine obtained from more conventional proton and phosphate MRS studies would also be useful (cf. section 3.5.1). A second example is based on the changes of intracellular calcium oscillations and extracellular calcium waves generated by neurotransmitter stimulated glia. These calcium signals stimulate glutamate release, modulate neuronal excitability and carbohydrate metabolism [86,89]. Changes in calcium concentration can be monitored by new contrast substances being developed for MRI investigations. These techniques, and recent applications of 2-photon optical calcium imaging in animals, could also address the role of dopamine D1 and D2 receptors [233] as well as metabotropic glutamate receptors and their modulation by α_1_-adrenoceptors in the stimulation of calcium responses in astrocytes [85,234,235]. A third example is the need for cross-sectional if not longitudinal MRS data on myo-inositol. This is suggested to be a marker of the integrity of glia and glial transport mechanisms, but the signal has proved difficult to separate from that for the large amounts of glycine present that also resonate at 3.6 ppm [236]. Whereas increased levels of myo-inositol are claimed to reflect gliosis that would not be expected in ADHD, decreased levels, as reported for major depressive disorder, may reflect glial loss or altered glial metabolism [237]. Indeed a functional decrease may apply to ADHD. A preliminary report on 15 subjects with ADHD found an increased glutamate/myo-inositol ratio [238], that would be consistent with either increased extracellular glutamate concentrations or an inadequate supply of myo-inositol.

#### 4.1.2 Origins of a putative energy and lipid deficiency

The principle claim of our hypothesis, that lactate production and availability is impaired, may be difficult to measure directly in human subjects, but may be best tested with a pharmacological challenge in animal models of ADHD such as the spontaneously hypertensive rat (SHR) [239,240] and poor (and impulsive) performers on the 5-choice serial reaction time task that show low 2-deoxyglucose uptake (index of brain glucose uptake) in the cingulate and ventrolateral orbital cortices during performance of the visuospatial task [241]. Lactate production should be recorded at baseline, during performance of the task, and after methylphenidate, atomoxetine or venlafaxin treatment (transport inhibitors of the three monoamines). The effect of pretreatment with monoamine receptor antagonists could also be investigated. The link between performance, energy availability and at least some of these treatments should be established. Other cognitive tests that make use of dynamic strategies (change in contingency) can be used to evaluate possible correlation between changes in lactate production/utilization and performance speed/accuracy.

Glutamate availability for uptake into astrocytes and stimulation of lactate production requires special attention. Impaired release would reduce astrocytic lactate synthesis. The impairment could lie with SNAP-25, a protein important for the release of the transmitter. There is some evidence for an association between polymorphisms of the SNAP-25 gene and ADHD [242,243]. A potential relationship to energy production should be investigated. Lactate synthesis is dependent on glutamate uptake into astrocytes. Problems with glutamate transport could give rise to deficient lactate synthesis. Metabotropic glutamate receptor function and the glutamate/aspartate transporter (GLAST) have been suggested to play a role the development of fatigue [78,244]. To test this hypothesis, glutamate metabolism could be measured in astrocytes of animal models of ADHD using an experimental setup that simulates the role of neurons (glutamate producers and glutamine consumers) by the addition of glutaminase to the culture medium [245]. A steady supply of glutamate can be imposed at the expense of glutamine, and the stress intensity manipulated by changing the glutaminase concentration [246]. The extracellular concentration of glutamate will provide information on the efficiency of flux through the glutamate transporter and glutamine synthetase system in restoring the extracellular concentration of glutamate to a low level [247]. This will provide a measure of the glutamate drive of lactate formation.

More quantification of developmental changes in myelination in ADHD, using sensitive neuroimaging techniques such as DTI, MRS, EEG or MEG would be informative. More simply, for indications of impaired white matter generation blood samples should be taken from healthy children and those with ADHD to investigate lipid availability (serum fatty acid levels and the nature of red blood corpuscle membranes). This approach has been useful in showing changes in patients with schizophrenia and depression (e.g. reduced polyunsaturated fatty acids like arachidonic and docosahexaenoic acid [246,247], decreased linoleic acid [248], less high-density lipoprotein levels [249]). An early report on hyperactive children suggested there were slightly decreased levels of poly-unsaturated, but increased levels of saturated fatty acids [250]. Chen et al. [251] found decreased nervonic acid, linoleic acid, arachidonic acid, and docosahexaenoic acid in red blood cell membranes of children with ADHD while plasma gamma-linolenic acid and red blood cell oleic acid were increased. Irmisch noted a potential relationship with stress experienced by their subjects [250]. As glucocorticoids modulate membrane stability and the adrenal hormone dehydroepiandrosterone influences lipid synthesis, both steroids should be monitored in the recommended study [252] (section 3.4.1.3). The availability of these fatty acids (particularly of the omega series), as well as marked prostaglandin synthesis (e.g. cyclooxygenase) around puberty are crucial determinants of synapse modification, pruning and maturation processes [253] that may be delayed in ADHD. These studies would usefully supplement further delineation of the fibre bundles that are [254] or in ADHD are not showing normal developmental increases of anisotropy studied with DTI.

Of the numerous potential genetic sources of the modification of energy availability, we would select those that regulate lactate metabolism (lactate, glutamate and glucose transporters, glycogen synthase, glycogen phosphorylase, glycolytic enzymes), factors that regulate glutamate release from synaptic terminals and hence glutamate-stimulated lactate production (e.g. SNAP25, vesicle transporter, metabotropic glutamate receptors, adenosine A1 receptors, neurexins, factors that regulate intracellular Ca^2+^), and astrocyte function (e.g. noradrenergic α_1_, α_2 _and β-adrenoceptors, dopamine D1 receptors). Mitochondrial function should also be considered, as impairment of gene transcription can occur through the accumulation of mitochondrial DNA mutations [255]. One potential candidate is Ant1, a mitochondrial ATP/ADP exchanger that facilitates efflux of ATP out of the mitochondria and glutamate uptake into astrocytes [256]. Metabotropic glutamate receptors are of special interest as stimulation can produce marked Ca^2+ ^oscillations. Its expression represents a glial sensor of the extracellular glutamate concentration that can be used to acutely regulate excitatory transmission [257].

Likewise, there are many genetic influences on lipid synthesis and myelin formation of potential relevance to our hypothesis on oligodendrocyte function in ADHD. We select but one for attention. Lingo-1 is expressed in oligodendrocytes and is critical for CNS myelin formation. Treatment of neuron and oligodendrocyte cell culture with soluble Lingo-1 (a transmembrane protein binding to the Nogo-66 receptor/p75 signaling complex) led to highly developed and differentiated axons [258,259]. In animal studies it appears that its up-regulation may be a characteristic of activity-induced neural plasticity responses [260]. Thus, its impaired expression could be a feature of several developmental disorders. We would also advocate the initiation of microarray studies of gene expression in samples taken from subjects with ADHD. This large scale profiling technique applied to patients with schizophrenia has already shown a surprising number of genes related to energy metabolism, oligodendrocyte function and myelin formation that are associated with psychopathology [261].

### 4.1 Neurophysiological and behavioural consequences of an energy deficiency

Neuroimaging techniques reflecting glucose or oxygen utilization would be well-suited to examine the effect of cognitive challenges on glycogen, glucose or lactate utilization (e.g. PET with deoxyglucose, near-infra-red spectroscopy of oxygenated/deoxygenated haemoglobin [262,263]). To what extent do task manipulations (event-rate, delayed reinforcement and difficulty) elicit differential responsiveness according to these measures in within-subject designs with symptomatic and asymptomatic individuals?

Similar task manipulations should be used during EEG monitoring of sustained neuronal firing and connectivity (EP latencies, theta/beta ratios and the coherence between sites of signal processing). Do people with ADHD show an early onset of fatigue or feel that they exert undue effort to overcome the deficiencies across a period of task performance? Skin conductance records would provide an independent reference for changes in the level of activation. However, analyses should carefully consider issues that could mask clear-cut associations such as the different types of oscillation pattern and the sub-diagnosis often encountered in an ADHD population [264,265]. Baseline measures should then be compared with the effects of medication and carbohydrate loading.

One test of energy deficiency would be based on the lactate and glucose dependency of the neurophysiological spike activity underlying LTP [266], the basis of short-term working memories often poorly expressed in ADHD (section 2.1). Animal work has shown that LTP, engendered in models of ischemic conditions, is amplified by dopamine D1 action on the AMPA (but not the NMDA) component of LTP [267]. Would ADHD subjects with a demonstrable lactate shuttle impairment show improved working memory following psychostimulant induced dopamine D1 stimulation of AMPA function?

A behavioural approach could also be applied. Under high cognitive demand (e.g. the incompatible conditions of asynchronous flanker tasks) energy requirements are high and stored resources limited. Mental effort and energy mobilization may be measured via blood glucose levels and cardiovascular function [268]. Even though the measures are relatively simple, they are yet likely to be associated with the variability of performance over time and be capable of modification by agents that modify monoamine transporter function. Support for this prediction derives from a report on two cases of spino-cerebellar ataxia. The authors describe a strong association between PET measures of decreased glucose utilization, SPECT measures of reduced dopamine transporter binding in the putamen and poor performance on tests of executive function [269].

## 5 Implications of the hypothesis

### 5.1 Implications for basic research

Although it is not possible to state which brain areas will be affected by restricted energy (lactate) supply, those with the highest energy demands are more likely to be affected and may be reduced in size [93]. In support of this implication of the hypothesis, numerous studies have found reduced brain volume in people with ADHD, particularly prefrontal cortex, cerebellum, corpus callosum, and basal ganglia, areas of the brain engaged in information processing and planning/selecting the appropriate behavioural response [102,103,147,270].

The hypothesis H1 implies that people with ADHD may develop strategies to overcome their inability to fully utilize the more efficient lactate shuttle during periods of high energy demand, i.e. extended high frequency neuronal firing. In order to avoid catastrophic depletion of localized energy reserves, a strategy may have evolved during development to avoid prolonged high-frequency firing of neural circuits by switching to other neural circuits that produce behaviours that may help to complete the task. When required to focus and concentrate on performing a specific task, their behaviour is automatically switched to other circuits. The advantage of this strategy may be that they explore the environment more actively and appear to learn more quickly initially, but they fail to learn new rules when the contingency changes because of their inability to stay on task. This may cause the individual with ADHD to appear easily distracted, because of rapidly switching from one task to another to avoid highly localized depletion of energy reserves. Evidence to support this suggestion is provided by clinical studies. In both Norwegian and South African populations of children with ADHD, Aase, Meyer and Sagvolden [8,9,271] demonstrated increased intra-individual variability of spatial location of motor responses, independent of accuracy of performance and time on task (greater spatial variability but not greater temporal variability because there were no external time demands placed on participants), suggesting that sensory-motor reflexes were not as efficient as in controls, possibly because of impaired energy supply to rapidly firing neurons in cortico-striatal and/or cortico-cerebellar circuits. Impaired maintenance of high neuronal firing rates due to suboptimal ATP production would impair learning at all levels of neural circuits involved in memory formation, including prefrontal cortex, striatum, cerebellum, and parietal cortex. Variability of spatial location would engage different neural circuits and may reflect a strategy developed to overcome the problem of insufficient lactate reserves to meet demands of prolonged rapid neuronal firing. Variability persists into adulthood [272,273]. The apparent remission of symptoms in many adults may be due to their elaboration of a set of redundant processing elements, and the neural procedures for their seamless engagement, which provide alternative task-relevant procedures lacking in children.

The hypothesis H1 implies that myelinated and unmyelinated axons will be affected differently. Non-myelinated axons are less efficient than myelinated axons and we predict that they may be more vulnerable to the lactate deficiency. Interestingly this would target non-myelinated nigrostriatal, mesocortical and mesolimbic dopamine projections, among others, which would affect the development of their target structures including striatum, prefrontal cortex and hippocampus. Compromised dopamine neuronal firing could account for decreased dopamine function in target areas as proposed by Sagvolden et al [20] in the dynamic developmental theory of ADHD. Drugs used to treat ADHD, such as methylphenidate, block the dopamine transporter and can thereby enhance the dopamine signal without requiring increased firing of dopamine neurons.

A further critical implication of the hypothesis H1 is that therapeutic drugs should directly or indirectly stimulate lactate production by astrocytes.

### 5.2 Implications for theories of ADHD

The current hypothesis extends existing theories of ADHD by proposing a physiological basis for specific aspects of the ADHD phenotype – namely frequent, transient and impairing fluctuations in functioning, particularly during performance of speeded, effortful tasks. We briefly point to the interfaces and overlaps.

One proposal attributes many features of ADHD to problems with the regulation of 'state' that affects the allocation of 'energy' and 'effort' [274,275]. Based on the "cognitive-energetic" model of Sanders [276] they define state regulation as the allocation of extra effort to defend performance in the presence of stressors such as high presentation rates of stimuli. Whereas CNS activation normally increases with event-rates, long inter-event intervals engender a sub-optimal hypoactivation in people with ADHD, who then are unable to recruit the necessary effort to adjust appropriately to the demands of the situation. Activation can be viewed as a state of readiness in selectively targeting possible outcomes of behaviour: The monitoring of input, endogenous interactions and feedback are involved. Effort is viewed as a process necessary for coupling and re-coupling input-information, neuronal modules and activational processes [277]. Our hypothesis is broadly consistent with this 'state-regulation' theory, but elaborates details of its potential physiological basis, and that in turn is specifically directed to account for intra-individual variability of performance on sustained demand. The distinction with our formulation lies with a) our emphasis on coping with high energy demands rather than a state of hypoactivation at low event-rates, and b) our prediction that the consequences of impaired lactate shuttle function lie with reduced rapid neuronal firing when that is required, and the delayed development of myelination. We do not predict a generalized 'state of activation' as described by Sergeant and van der Meere [274,275].

Barkley (1997) considers symptoms of ADHD to arise from the disruption of neurocognitive control. Like Barkley we have selected top-down, executive processes for special but not exclusive consideration. We emphasise intra-individual variability in the control of the maintenance or switching of set appropriate to adaptive planning, and behavioural organization towards reasonable goals. Barkley, however, views these brain-behaviour relations to be mediated fully by inhibitory processes. Our hypothesis is not so restrictive, and thereby questions the centrality of inhibitory processes to ADHD. What Barkley sees as inhibition we see as a failure of activation, for which we provide a specific mechanism.

Our hypothesis overlaps with the explanations offered by Sonuga-Barke [278] and Sagvolden et al. [20]. Sonuga-Barke's *dual pathway theory *invokes a role for the mesocortical dopamine system in modulating (deficient) dorsal fronto-striatal glutamatergic mediation of some executive functions. It also envisages a role for the mesolimbic dopamine system in the anomalously functioning reward and motivation-influencing circuits of the more ventral frontal-accumbens glutamatergic system [107]. The mesolimbic role is often associated with the preference of ADHD subjects for immediate rather than delayed rewards. This relates to the dynamic developmental theory of Sagvolden et al., which concentrates on the registration of reinforcement and related motivational consequences, in particular, a mesolimbic impairment of the "efficiency with which the contingency between present action and future rewards is signalled". In ADHD research there is widespread agreement that there is a reduction in the control by future rewards of current behaviour, and an increase in the extent to which they are discounted (i.e., a steeper delay of reward gradient). Sagvolden et al. also extend their theory to impaired function of the ascending nigrostriatal and mesocortical dopamine pathways.

Both theories are driven by interpretations of the relative success of the psychostimulants in terms of dopamine function as a neurotransmitter, and the need to explain responsivity to reinforcement in ADHD in a wider motivational context. To a degree the explanations are successful. But we would shift emphasis in our interpretation. The first shift is clearly from a neurotransmitter interpretation of psychostimulant function to the additional role of glial stimulation. The second shift concerns the emphasis on the role of delay periods. Our hypothesis views these as requiring the operation of attentional and working memory components of executive function. Typically such delays require consistent firing of prefrontal neurons with high demands on energy resources [279]]. To a degree our emphasis on impaired function under high demands is consistent with Sonuga-Barke's description of impaired efficiency of mesocortical dopamine control of fronto-striatal circuits, and Sagvolden's description of the difficulty for children with ADHD to comply with (the demanding) shallow reinforcement gradients. According to our hypothesis, the reinforcement contingency is not so much a causal factor in ADHD, as it is an occasion in which the mechanisms we have outlined are likely to be operative. People with ADHD will tend to go "off-task" independent of the reinforcement lost because the effort to remain "on-task" requires energy resources that are no longer available to them.

### 5.3 Implications for ADHD in the clinic

Impaired performance is expressed mainly in tasks of high temporal and/or cognitive demand and will be less apparent in self-paced tasks of low complexity. Proposals for modifying current treatment methods can be made at the behavioural and the biochemical level. Segmenting tasks to reduce concurrent demands for speed and accuracy of responding would be helpful in situations that place high and prolonged demands on neural activity (function). One possibility is to introduce frequent variation into the nature of the task or its modality in order to allow for a re-distribution of the energetic demands to alternate neural processing circuitry. A second possibility is to introduce frequent breaks into ongoing, lengthy and demanding activities such as school exams, assembly lines and motorway driving. Both strategies would permit energetic demands to be distributed to alternate resources and the re-establishment of depleted energy reserves. A third method is the institution of self-paced rather than system-paced scheduling of the required activity. Each strategy would reduce concurrent demands for both the speed and accuracy of performance during continuous and complex information processing tasks. Also, if speed and accuracy are required in a complex task, a third possibility is to reduce the cognitive demands by segmenting the task into smaller and simpler steps, with breaks between steps.

Investigations of potential therapeutic agents that target the energetic system rather than neurotransmitter function may yield additional improvements in treatment beyond the positive modulation of the energy balance noted for monoaminergic drugs in current use. One aim could be to stimulate creatine kinase function, and thus to increase the availability of phosphocreatine, and the re-synthesis of ATP. Increasing creatine levels would have the additional advantage of being neuroprotective [280]. While there is clearly a need to restore carbohydrate and energy reserves and to ensure an adequate supply of omega fatty acids and other elements essential for myelin synthesis, it is equivocal whether dietary supplementation is capable of delivering the requisites to where they are needed. In the longer term it may prove more feasible to modify the expression of genes demonstrated to be essential in the regulation of astrocyte function, lactate production, glutamate transport and myelin synthesis [261]. Several novel forms of therapeutic agent and delivery system based on the components of the energy cycle and the synthesis of myelin could be tested.

## 6 Abbreviations

Acetyl CoA acetyl coenzyme A

ADHD attention-deficit hyperactivity disorder

ADP/ATP adenosine di-/tri-phosphate

AMPA alpha-amino-3-hydroxy-5-methyl-4-isoxazolepropionic acid

Ca^2+ ^calcium ions

CNS central nervous system

CNV contingent negative variation

CPT continuous performance test

DTI diffusion tensor imaging

EEG electroencephalogram

EP evoked potential

ERP event-related potential

FA fractional anisotropy

GLAST glutamate/aspartate transporter

GLT-1 glutamate transporter

Glu glutamate

^1^H-MRS proton magnetic resonance spectroscopy

ISD individual standard deviation

K^+ ^potassium ions

MRI magnetic resonance imaging

MRS magnetic resonance spectroscopy

MRT mean reaction time

Na^+ ^sodium ions

NAA N-acetyl-aspartate

NMDA N-methyl-d-aspartate

Phe phenylalanine

PKU Phenylketonuria

RT Reaction time, or response time

SD standard deviation

## 7 Competing interests and authors' contributions

The authors have no competing interests and are listed in approximate order of individual contribution to the manuscript. The first four authors were the major contributors, VA Russell, RD Oades, R Tannock, and PR Killeen followed by JG Auerbach. All authors contributed to discussions and helped to draft the manuscript.
